# Functional Characterisation of *GmGASA1-like* Gene in *Glycine max* (L.) Merr.: Overexpression Promotes Growth, Development and Stress Responses

**DOI:** 10.3390/life14111436

**Published:** 2024-11-06

**Authors:** Mohamed A. S. Khalifa, Qi Zhang, Yeyao Du, Nooral Amin, Baozhu Dong, Piwu Wang

**Affiliations:** 1 Centre of Biotechnology, Jilin Agricultural University, Changchun 130118, China; mohamed.said@agr.cu.edu.eg (M.A.S.K.); zhangxiaoqi6969@163.com (Q.Z.); du8158152022@163.com (Y.D.); aminagric965@gmail.com (N.A.); 18347513054@163.com (B.D.); 2 Faculty of Agriculture, Cairo University, Giza 12613, Egypt

**Keywords:** *Glycine max*, *GmGASA1-like*, gene functions, yield, plant architecture, abiotic stresses

## Abstract

The presence of *Gibberellic Acid*-*Stimulated Arabidopsis*, *GASA*, gene family has been reported in many important plants, playing roles in various aspects of plant biology but little has been uncovered in soybeans. Soybean is one of the major plants providing nutrition for humans and livestock globally. In this study, we overexpressed a novel *GASA* gene (*GmGASA1-like*) in *Glycine max* and conducted bioinformatic analyses, evaluated the T2 transgenic line in an open field, and applied major stressors along with the growth promoter GA_3_ to investigate the potential functions of the *GmGASA1-like* gene. The results of bioinformatics implied that the *GmGASA1-like* gene is regulated by GA_3_, and its protein has the potential to influence key processes of plant growth and development. The transgenic plants (JN74-OE) were significantly taller and had a larger canopy in the field trial at the R1-growth stage and demonstrated superiority in some seed quantity and quality traits after harvesting. Under abiotic stresses (including cold, heat, and drought) and spraying of GA_3_, the level of *GmGASA1-like* gene expression in JN74-OE exceeded the levels measured before the treatments. Notably, the highest expression level was observed during the drought stress treatment. Photosynthesis pigments levels in both the overexpressed lines and the control group showed no significant differences. In summary, this study sheds light on the multifaceted roles of the *GmGASA1-like* gene, impacting soybean plant architecture, seed traits, and stress responses. Ultimately, this research paves the way for a more productive and sustainable soybean industry.

## 1. Introduction

Soybean (*Glycine max* L.) is a key member of the Fabaceae family, cultivated in various climates, including tropical, subtropical, and temperate [1] regions. Soybean seeds contain about 20% vegetable oil [2], and they account for 55% of global vegetable oil production [3], offering high-quality oils with excellent thermal stability, low saturated fat, and are rich in essential fatty acids, particularly oleic acid and vitamin E [3,4]. Additionally, soybeans have a protein concentration of around 40% dry weight, surpassing other legumes by 20–37% [5,6]. Despite their oil and protein content, market value is primarily driven by meal fractions, with soybeans supplying 70% of the world’s protein meal consumption in 2018, totalling 235.4 million metric tons [7]. As global demand for soybean oil and protein continues to rise, increasing soybean production is crucial for securing food supplies [8]. Consequently, developing new high-yield varieties remains a key goal for soybean breeders [3]

Over 46,430 protein-coding genes from the soybean genome could be predicted by sequencing, most of which lack functional analysis at present [9,10]. Bioinformatics has revolutionised the understanding of gene functions by providing powerful computational tools and analytical methods which contribute to gene discovery and functional annotation. Tools like SWISS-MODEL and Phyre2 predict 3D structures from amino acid sequences [11]. Bioinformatics maintains curated databases (e.g., UniProt, Gene Ontology, KEGG) that provide functional annotations for genes and proteins [12]. From analysing DNA sequences to comparing genomics, [13], available resources in this aspect make breeding programs and gene function studies more straightforward. 

The *GASA* gene family is a unique set of genes in the plant kingdom, and most of their members are regulated by gibberellins [14,15]. The *GASA* genes generally encode small proteins with three distinct domains: a single peptide domain in the N-terminal sizing between 18–29 residues, a variable region in both length and peptide chain sequences (7–31 polar residues), and the third domain is a segment of approximately 60 amino acids incorporating a conserved domain of 12 cysteine residues named GASA at the C-terminals [16,17]. In plants, GASA proteins were speculated to be related to various development processes, such as cell division and organ development [18]. Also, gene expression behaviour widely distributed in different plant parts strengthens the possibility of wide roles of these genes in cell growth and differentiation [19]. To date, 15 *Snakin*/*GASA* genes have been identified in *Arabidopsis*, 9 in rice, 10 in maize, 16 in potato, 26 in apple, and 38 in cotton [17,20]. In *Arabidopsis*, *AtGASA5* was found to have a role in controlling flowering time and stem development [18]. In cotton, *GhGASA* genes possibly participate in fibre development [21]. In strawberry, *FaGAST2* gene overexpression led to the production of smaller fruits with smaller parenchymal cells [22]. In apple trees, *GASA* family members were observed to affect the flowering event [17]. *HbGASA7*-1 and *HbGASA15* play important roles in the early stage of resistance to *Colletotrichum gloeosporioides* in rubber trees [18]. *GsGASA1*, from *Glycine soja*, has been found to inhibit root growth under chronic cold stress [23]. Information on the *GASA* gene family in soybeans is limited. A genome-wide study has identified 37 *GASA* gene members in the soybean genome. While many of these genes have been studied in other organisms, further systematic research is necessary to understand the structure and biological functions of *GmGASA* genes in soybeans [24]. This study aims to explore the role of the *GmGASA1-like* gene, a novel member of the *GASA* gene family, through bioinformatic analysis and evaluation of the growth, productivity, and performance of the JN74-OE plants under various stress conditions and GA_3_ spraying.

## 2. Materials and Methods

### 2.1. Cloning and Characterisation of GmGASA1-like

RNA was extracted from the first leaf of the trifoliate soybean cultivar Williams 82 (seed materials were provided by the Centre of Biotechnology, Jilin Agricultural University) using a RNAiso Plus (Cat. #9109) kit, TAKRA Bio, Shiga, Japan. cDNAs constructions were conducted by All-in-OneTM First-Strand cDNA Synthesis Kit (Cat. No. QP006). The *GmGASA1-like* gene (ID: Glyma.06G024500 (PAC:30550949)) information was collected from the Phytozome database. Transcriptional sequence (706 bp), Supplementary Section S1, was hunted by (*S: 5′-GCATACTGAAGAGTGAAGAG-3′, AS: 5′-TAGCAACACAACAAATTAAG-3′*) primers and 2 × Es Taq MasterMix (Dye), CW0690S CWBIO, China. PCR reaction: initialisation at 95 °C for 5 min, 35 cycles of denaturation at 95 °C for 40 s, annealing at 54 °C for 40 s, extension at 72 °C for 40 s., and final elongation at 72 °C for 8 min before holding and saving at 4 °C. The amplified products were electrophoresed by a gel electrophoresis system (BioVolt 300V with 22 cm × 12.5 cm). A 1× TBE buffer was used as a running buffer. *Trans2K*^®^ and *Trans5K*^®^ (Transgen Biotech Co., Beijing, China) were used as DNA Markers. The run time was 25 min at 180Volt and 90Amp. BioDoc-It^®^ Imaging, UVP^®^, was used for documentation. DNA recovery was achieved using the AxyPrep DNA Gel Extraction Kit, Axygen Scientific, Inc, Beijing, China. The quality test was performed using the NanoDrop 1000^®^ Spectrophotometer, Thermo Fisher Scientific, Shanghai, China. High-quality purified-specific cDNA for the targeted gene was used for cloning to the pMD-18T vector and then to the plant overexpression vector (pCAMBIA3301) by a Seamless Cloning Kit from Clone Smarter Technologies^TM^ (C5891-25). Further details are provided in Supplementary Section S1. 

The data generated from the Phytozome database, PLAZA database, and PROTTER were used to describe the gene structure. The physical and chemical properties of the protein were studied through Expasy, ProtScale, NetPhos-3.1, SOPMA model, and AlphaFold. Subcellular localisation predictions were made by SignalP 3.0., TMHMM-2.0 server, and ProtComp Version 9.0. Phylogenetic tree analysis was performed using the NCBI database based on the protein amino acid sequence. MEGA11 (V.11.0.13) software was used to construct an unrooted phylogenetic tree using the neighbour-joining (NJ) method, and bootstrap analysis was performed with 1000 replicates [25]. Promoter analysis was conducted using the PlantCARE tool, and results were drawn using Tbtools software (V.1.110). Links for all the used tools are available in Supplementary Section S2.

### 2.2. Genetic Transformation and Detection 

Soybean seed materials of the JN74 variety were used as the recipient for *Agrobacterium*-mediated transformation in a tissue culture system, utilising cotyledonary nodes from five-day-old seedlings as explants [26,27]. The T0 plants obtained from tissue culture were cared for in a greenhouse until their seeds were collected. The collected seeds from the T0 generation were sown in a natural field for testing, and sufficient seeds from the T1 generation were gathered for subsequent experiments in the T2 generation. Leaf samples for DNA extraction and detection of transformed plants of the T1 generation were collected after one month of the T1 generation planting. DNA extraction was accomplished by NuClean Plant Genomic DNA Kit (CW0531) CWBio, China. Transgenic plants were detected by screening for the nopaline synthase (NOS) terminator and Cauliflower Mosaic Virus (CaMV) 35s promoter, which flank the gene of interest in the pCAMBIA3301 plant expression vector. Primers are listed in Supplementary Section S3.

### 2.3. Field Performance and Yield Evaluation 

#### 2.3.1. Seed Materials Planting

The transgenic T2 generation and control materials were planted in the experimental base of Jilin Agricultural University (43°88′ N, 125°35′ E) in May 2023. The experimental site has a temperate semi-humid continental monsoon climate with 500–600 mm of water rain precipitation, an annual average temperature of 5.8 °C, thermal accumulation of 2850 °C per year, and a frost-free period from May to the end of October. The soil type is classified as meadow soil, and four metal protective fences surrounded the experimental site. Seeds were grown in rows (0.65 m wide by 2.0 m long), with a plant spacing of 0.08 m in a row.

#### 2.3.2. Growth and Yield Parameters Measurements

At the beginning of flowering (R1-growth stage), JN74-OE at T2 generation and control plants (JN74) were evaluated for the following growth parameters: plant height, number of nods per plant, trifoliate leaf length, trifoliate petiole length, parameters for leaflets, and plant canopy. At the end of the season, the plants were harvested, and measurements for plant height, number of nodes per plant, number of pods per plant, seed weight per plant, and 100-seed weight traits were recorded. The oil and protein content, in addition to seed fatty and amino acids in JN74-OE and control, were determined using NIRS DS2500 near-infrared analysis instrument. Briefly, the instrument used cleaned whole seeds to evaluate the aforementioned seed traits. 

### 2.4. Application of GA_3_ Spraying, Abiotic and Biotic Stressors

Seeds of JN74-OE and the control plants were grown in small pots with an 8 cm diameter, filled with sterilised soil mixture (peat moss + perlite + sand in a ratio of 2:1:1). Pots were seated in trays in which each tray contained 18 pots and about 36 plants in total. All trays had nine pots for control and nine pots for JN74-OE seeds. One tray was used to conduct one of the following stress treatments: cold, heat, salt, drought, or GA_3_ treatment. The plants were kept in a growth room under these controlled environmental conditions: 15–23 °C, 60% relative humidity, and 16 h light/8 h dark cycles. After one month, different stresses and GA_3_ treatment were applied. 

To induce cold stress, the plants were subjected to a temperature of 4 °C for 24 h, while for heat stress, they were exposed to 42 °C for the same duration. For both treatments, 80% RH and light intervals of 16 h light/8 h dark cycle were provided in the growth chamber (HDL apparatus/HPG-280HX) [28]. For salt stress, 250 mM NaCl solution was used as an irrigation dose. For drought stress, the plants were left without irrigation till the symptoms of thirst were obvious on them [29]. For GA_3_ treatment, GA_3_ solution was prepared in a concentration of 0.1 g/L. Afterwards, 200 mL of it was sprayed over one tray of plants [30]. Leaf-tissue samples were collected from the treated plants and saved at −80 °C for further RNA extraction. In the case of spraying GA_3_, samples were collected 24 h after cold, heat, and salt treatments. In contrast, samples from drought-stressed plants were collected after observing symptoms of dryness on the leaf margins.

Culture from the *Cercospora sojina* fungus in a petri dish was provided by the plant protection laboratory at JLAU. Conidial suspensions were prepared by flooding fungus colonies with sterile water and gently scraping them. The suspensions were filtered through multiple layers of cheesecloth and then sprayed over one tray of plants. For relative humidity at a high level, the inoculated plants were covered with clear plastic bags for 48 h. Disease symptoms were evaluated after one month as a qualitative feature (sensitive versus resistant) [31]. The soybean mosaic virus (SMV) was provided by the plant protection laboratory at JLAU in the form of old soybean-infected leaves. Inoculum leaves were well crushed in phosphate buffer (0.1 mol/L, pH 7.0) by using a sterilised pestle and mortar. A small amount of carborundum was mixed with the virus solution, and then, by using a small brush, plants in one tray were inoculated. Virus infection symptoms were evaluated after one month as a qualitative feature (sensitive versus resistant) [32]. 

### 2.5. Quantitative Real-Time PCR Assay 

Total RNA was isolated using *TransZol* UP and then treated by Dnase I (Rnase-free), both from the TransGen Biotech, Beijing, China. The isolated RNA was then subjected to reverse transcription using the SureScript™ First-Strand cDNA Synthesis Kit, GeneCopoeia, Guangzhou, China. Quantitative real-time PCR was performed on each cDNA sample using a SYBR Green-based mix, the All-in-One™ qPCR Mix from GeneCopoeia, Guangzhou, China. An Agilent-MX3000P q-PCR system was used to run the quantification. The NCBI primer design tool was used to generate primers, and the successful ones for the *GmGASA1-like* gene were used (Supplementary Section S4). Soybean *β-tubulin* (*Glyma20g27280*) gene was used as a reference gene to normalize the expression levels of the target gene. The comparative CT method (2^−ΔΔ^CT method) was used to analyse the data in Microsoft Excel [33]. 

### 2.6. Chlorophylls and Carotenoids

Leaf specimens were crushed with a pestle and mortar under liquid nitrogen condition; a total of 0.15 g was inserted in 25 test tubes containing 15 mL of 95% ethanol and then kept inside the dark at 4 °C in a refrigerator overnight. Chlorophylls and carotenoids were measured using Shimadzu Uvmini-1240 spectrophotometer, according to the method of Lichtenthaler and Buschmann [34]. 

All experiments were replicated, and comparisons were made using Microsoft Excel V. 2016 and IBM SPSS Statistics V.25 software. The figures were illustrated by Microsoft Excel 2016 and Originlab software V. 2018. 

## 3. Results

### 3.1. Characteristics of GmGASA1-like Gene Sequences

#### 3.1.1. Physical and Chemical Properties

**Figure 1:**
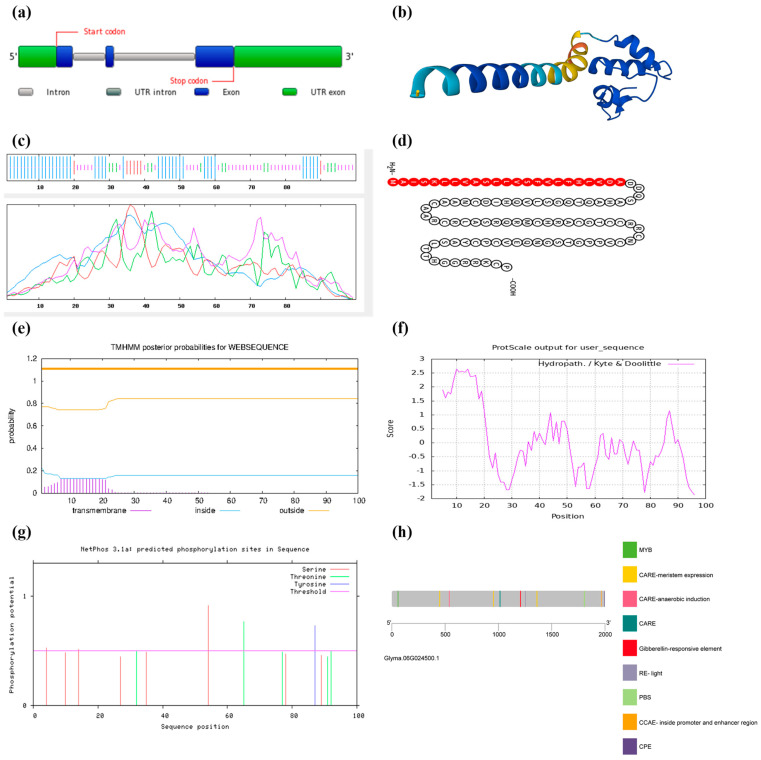
Structure of the *GmGASA1-like* gene and its protein. (**a**) Gene structure illustrated from Dicots PLAZA 5.0. UTR: the untranslated region and the three blue exons are the code sequences for the final protein amino acids. (**b**) Predicted 3-D structure from AlphaFold-MODEL which is dominated by the alpha helices, whereas the SOPMA model in (**c**) Predicted the following four conformational states: alpha helices, random coils, extended strand and beta-turn. (**d**) Peptide chain prediction from PROTTER tool; the red segment represents the signal peptide portion which agrees with the prediction of transmembrane domain from TMHMM-2.0 server (**e**). (**f**) Hydropathicity scale computed by ProtScale. Scores above zero refer to protein hydrophobic portions, while scores below zero indicate protein hydrophilic portions. (**g**) Sequence- and structure-based prediction phosphorylation sites. The horizontal line between 0 and 1 on the *x*-axis refers to the threshold of possible phosphorylation sites (**h**) Promoter unique elements. Data were retrieved from PlantCARE web tools, and unique promoter elements were illustrated by TBtools software (V.1.110).

The data generated from the Phytozome database (Wm82.a2.v1, sequencing edition), the PLAZA database for gene structure (Figure 1a), and the PROTTER tool for *GmGASA1-like* gene, showed that the gene sequence is 1249 bp, the transcription sequence is 706 bp, the coding sequence is 303 bp, and the protein sequence is 101 amino acids (Figure 1d and Supplementary Section S1).

Structural analyses provide crucial information about form and domain structure, protein classification, function prediction, and interactions with other biomolecules [35]. From Expasy, the GmGASA1-like protein has a molecular weight of 10,748.46 Dalton. The molecular formula for the expected protein is C_445_H_732_N_148_O_134_S_14,_ resembling 1473 atoms in total. The isoelectric point (pI) is 8.83, which indicates the existence of a high number of basic amino acids constructing the protein. The ProtScale score refers to a large proportion of the protein to be hydrophilic, as shown in Figure 1f, where most of the GmGASA1-like protein amino acids are below the score of zero. The instability index (II) is computed to be 40.62, which classifies the protein as an unstable one. A protein kinase is a kinase that selectively modifies other proteins by covalently adding phosphates to them in a process called phosphorylation. This results in a functional change of the target protein (substrate) by altering enzyme activity, cellular location, or association with other proteins [36].

Data of NetPhos-3.1 from DTU services [37], Figure 1g, show that the protein has three types of P-sites. Serine (S) locations include three repeats, one threonine (T) location, and one tyrosine (Y) location. From the SOPMA model, the secondary structure of the GmGASA1-like protein has four state components, as shown in Figure 1c, which are dominated by 42% alpha helices and 38% random coils. In contrast, the extended strand was 7%, and the beta-turn was 13% [38]. A Snakin-2 AlphaFold DB model of C6SWR9_SOYBN protein from *Glycine hispida*, with a sequence identity score of 82.83%, was identified as the top template for homology-based 3D modelling of the GmGASA1-like protein, as shown in Figure 1b.

#### 3.1.2. Subcellular Localisation Prediction 

Proteins are only allowed to enter the secretory system in prokaryotic and eukaryotic cells if they include a specific targeting signal known as a signal peptide (SP) [39]. SP prediction of GmGASA1-like protein was completed using SignalP 3.0. and the TMHMM-2.0 server [40]. Signal peptide probability was 1.000, and the maximum cleavage site probability was 0.992 between amino acids at positions 23 and 24, governing through a secretary pathway to the cellular membrane, as shown in Figure 1e. ProtComp Version 9.0 identified the subcellular location of the GmGASA1-like protein, possibly a multi-located protein, with a high likelihood of being extracellular.

#### 3.1.3. Promoter Analysis

Interaction between genes can be observed through promoter analysis for genes under study [41]. Data retrieved from PlantCARE web tools [42] revealed that a total of 147 CAREs were identified within 2 kb upstream of the start codon (ATG). Three light-responsive elements, such as one MRE, one G-box, and two GT1-motifs, were predicted in the promoter. Many stress-responsive elements were predicted, such as the anaerobic-responsive element (ARE), the MYB recognition sites involved in drought and ABA signals, as well as the MYC recognition site involved in drought, ABA, and cold signals. Furthermore, hormone-responsive elements, such as the gibberellin-response element (P-box), which responses to gibberellins in a way to modulate downstream processes (Figure 1h), were found in the promoter sequence. 

#### 3.1.4. Phylogenetic Tree and Protein Classification

**Figure 2:**
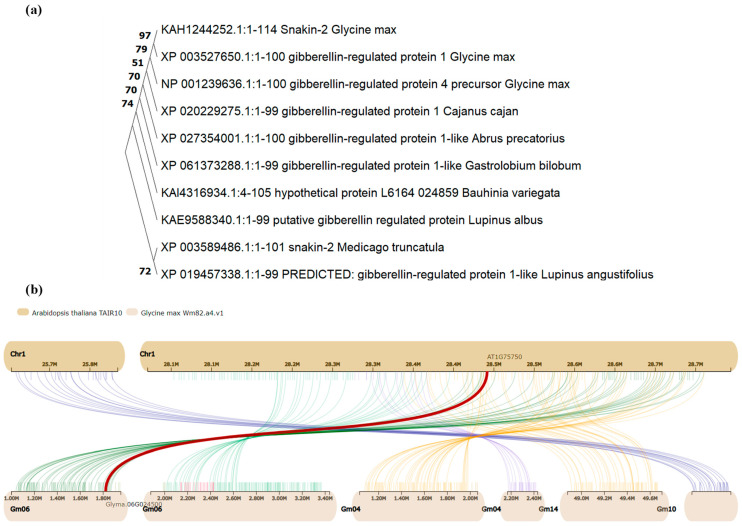
Sequence homology for the *GmGASA1-like* gene and its protein. (**a**) Phylogenetic tree. The protein peptide sequences were blasted into the NCBI database and the ten most relative sequences were used to draw the tree by MEGA11 software. Numbers over the main branches refer to the statistics of similarity. (**b**) Synteny with the nearest gene from *Arabidopsis* excavated from Phytozome. The *AT1G75750* on chromosome 1 from *Arabidopsis* is the most relative one to the *GmGASA1-like* gene on chromosome 6.

GmGASA1-like protein peptide sequences were obtained from Phytozome and blasted into the NCBI database using the default parameters. Then, the most relative sequences based on E-value were retrieved for constructing the phylogenetic tree, which was drawn by MEGA11 (V.11.0.13) software, as shown in Figure 2a [25]. According to the InterPro and Pfame databases for the classification of protein families, GmGASA1-like protein is predicted to be a member of the gibberellin-regulated protein family (GASA), under entries IPR003854 and PF02704. According to the PANTHER knowledgebase, GmGASA1-like protein is a member of Extension Proline-Rich Protein (PTHR23201) family and subfamily of Gibberellin-Regulated Protein 1-Related (PTHR23201:SF2). By using the Phytozome Synteny function, the TAIR10 (*AT1G75750*) gene at chromosome 1 from *A. thaliana* is the nearest one to the soybean *Glyma.06G024500* gene, as shown in Figure 2b. Arabidopsis gene, *AT1G75750*, is described in the TAIR database as *GA-responsive GAST1* gene homolog regulated by BR and GA antagonistically, possibly involved in cell elongation. 

### 3.2. Cloning, Transformation and Detection of Transgenic Plants 

**Figure 3:**
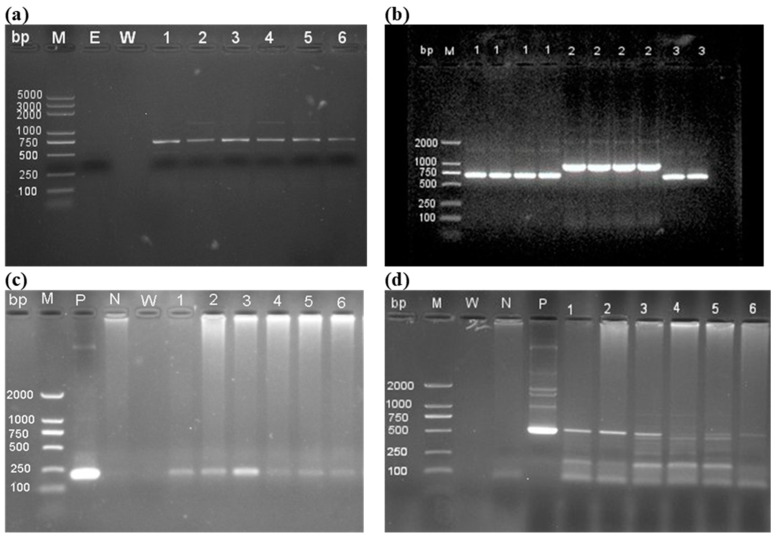
Cloning and detection of transgenic plants. Lanes M—DNA marker; W—water; E—PCR reaction without DNA template; lane P—positive control (pCAMBIA3301vector); lane N—non-transgenic plant. 1–6—transgenic plants (common across (**c**,**d**) panels). (**a**) Isolation of the *GmGASA1-like* cDNA, 706bp. Lanes 1–6: gene of interest. (**b**) Transformed *E. coli* by pMD™18-T- *GmGASA1-like* vector. Lane 1: Positive single colonies for the targeted gene, lane 2, and lane 3 non-targeted genes. (**c**) NOS terminator detection in transformed plants, T1 generation (target fragment is 192 bp). (**d**) CaMV35S promoter detection in transformed plants, T1 generation (target fragment is 500 bp).

*The GmGASA1-like* ORF CDS of 706 bp was successfully isolated from the soybean cultivar Williams 82, as shown in Figure 3a. Following this, the targeted bands were purified and cloned into the pMD™18-T vector, resulting in positive colonies (Figure 3b) that were subsequently sequenced by Comate Bioscience Co., Ltd., Changchun, China. The sequencing results, along with transcription data from Phytozome (*Glyma.06G024500*), were analysed using DNAman (V.6.0.3.9) software. The successful sequences were then employed to construct the plant overexpression vector pCAMBIA3301-*GmGASA1-like*, which can be found in Supplementary Section S1.2. The transgenic plants in the T1 generation were obtained by detecting the nopaline synthase (NOS) terminator and the Cauliflower Mosaic Virus (CaMV) 35S promoter, yielding bands of 192 bp and 500 bp, respectively (Figure 3c,d).

### 3.3. Effect of GmGASA1-like Gene Overexpression on Soybean JN74 Performance

#### 3.3.1. Evaluation of Growth and Yield Parameters at R1-Growth Stage and After Harvest

**Figure 4:**
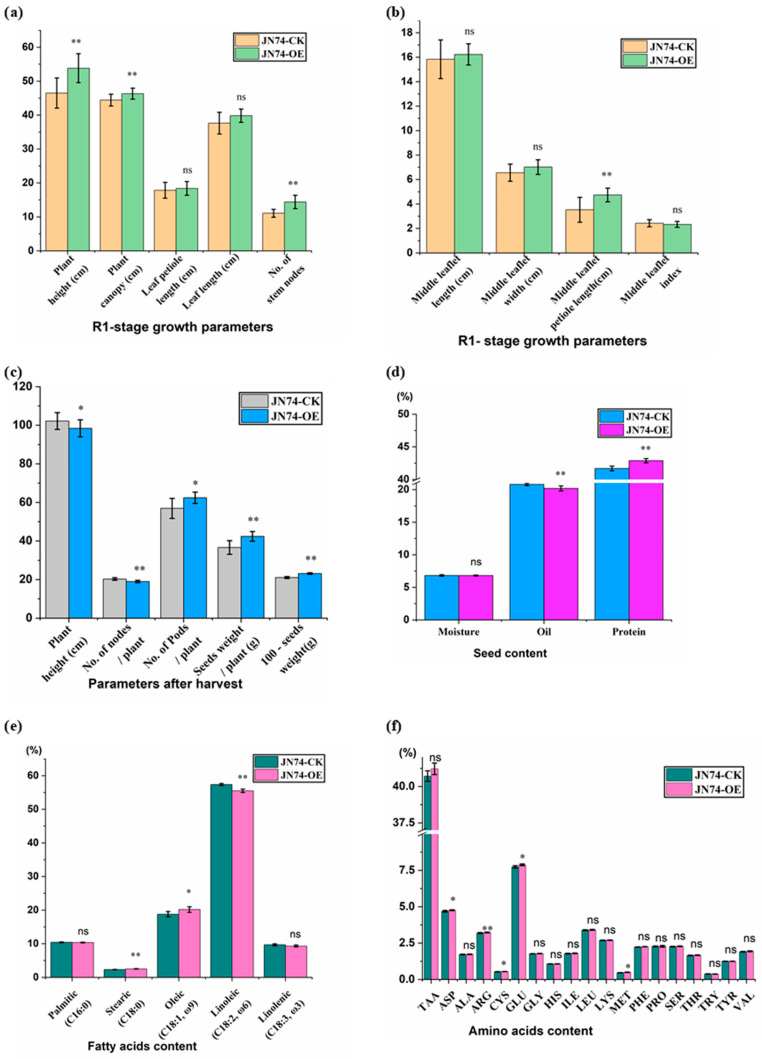
Evaluation of growth and yield parameters for JN-74-OE vs. CK. (**a**,**b**) Growth parameters at R1-growth stage, (**c**) Yield parameters after harvesting. (**d**) Seed moisture, oil and protein content. (**e**) Fatty acids content. (**f**) Seed amino acids content. Seed chemical properties were obtained by using the NIRS DS2500 near-infrared analysis instrument. The results are expressed as means of 11 readings ± standard deviations, and significance was obtained from paired samples *T*-test. The “ns” refers to non-significant changes, while “*” indicates significance (* *p* < 0.05; ** *p* < 0.01).

Data in Figure 4a–c present results from paired samples *T*-test for some vegetative growth and yield parameters collected at the beginning of flowering (R1-growth stage) and at the end of the season. Data show that the plant height, number of stem nodes, plant canopy, and middle leaflet petiole length traits in the JN74-OE are significantly greater than those of the control. On the other hand, leaf petiole length, leaf length, middle leaflet length, and width traits were changed slightly but without significance, as shown in Figure 4a,b. The maximum of plant height trait and number of nods per plant trait demonstrate a significant decrement in JN74-OE compared to the control; meanwhile, the number of pods, seeds weight per plant, and the 100-seed weight traits significantly increased. The seeds’ weight per plant trait shows superiority for the overexpressed line over the non-transformed line by an average of 5.82 g for plant yield, as shown in Figure 4c. 

#### 3.3.2. Seed Oil and Protein Assessment

Protein and oil content, as well yield quantity, are agronomically important traits that essentially account for the economic value of soybeans. The NIR spectrometer, Foss NIRS DS2500, was used to analyse the seed traits. Data in Figure 4d show paired samples T-test results for seed moisture, oil, and protein content in JN74-OE against control. While seed oil content decreased in JN74-OE by 3.02%, seed protein content increased by 2.87%. The physical, chemical, and nutritional values of the oil come from the ratio and amount of saturated and unsaturated fatty acids [4]. Data in Figure 4e show paired samples t-test results for seed fatty acid content in the overexpressed lines against the non-transformed lines. Palmitic and linoleic acids showed non-significant changes. While stearic acid rose significantly in the seed oil of JN74-OE by 9% and oleic acid by 7.39%, linoleic acid decreased significantly by 3.25%. Protein amino acid composition in soybeans and animals includes the following nine standard necessary amino acids: isoleucine, leucine, lysine, histidine, methionine, phenylalanine, tryptophan, threonine, and valine, which is why soybean protein is gaining popularity [43]. Data in Figure 4f show the results of paired samples *T*-test for seed amino acids content in JN74-OE against control. Amino acids in both JN74-OE and control are almost stable with no significant changes except for ASP, ARG, CYS, GLU, and MET, which show significant increases in the JN74-OE compared to the control, with increments of 0.84%, 1.8%, 1.85%, 1.14%, and 8.3%, respectively. Interestingly, the MET amino acid, one of the key points in evaluating soybean protein quality, recorded the highest increment.

### 3.4. Application of Stresses and Relative Gene Expression 

#### 3.4.1. Relative Gene Expression Patterns in Height-Diverse Soybean Germplasms

**Figure 5:**
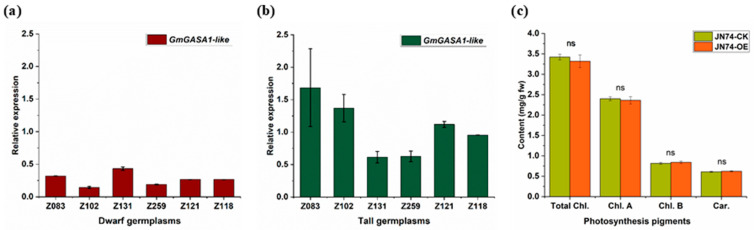
Relative expression of the *GmGASA1-like* gene in height-diverse soybean germplasms, and photosynthesis pigments in JN74-OE vs. CK. (**a**) Expression of the *GmGASA1-like* gene in dwarf germplasms. (**b**) Expression of the *GmGASA1-like* gene in tall germplasms. Z letters in figures a and b refer to the names of germplasms assigned by the Biotechnology Centre of Jilin Agriculture university. Soybean *β-tubulin (Glyma20g27280)* gene was used as a reference gene. The average ΔCt of all samples within the group (dwarf/tall germplasms) was used as a calibrator for individual samples to analyse fluctuations in *GmGASA1-like* gene expression within that group. Error bars represent standard deviation; n = 3. (**c**) Photosynthesis pigments determination in the JN74-OE vs. CK. Trait abbreviations: Total Chl.—total chlorophylls; Chl. A—chlorophyll A; Chl. B—chlorophyll B; Car.—total carotenoids.

To discover the correlation between expression variation of *GmGASA1-like* gene and plant height trait, different germplasms of soybeans were selected. The chosen germplasms have phenotypic differences in the plant height trait. Seed materials were grown, and samples were collected at the V1 growth stage (the first true leaf is fully unfolded) to determine the relative expression patterns through RT-qPCR. Data presented in Figure 5a,b indicate the high possibility of an existent relation between the plant height trait and the *GmGASA1-like* gene. That could be expected from the fluctuation of the relative expression between the soybean accessions that are phenotypically different in the plant height trait in addition to the existence of statistical significance between the plant height and the gene expression, as presented in Supplementary Section S5.

#### 3.4.2. Chlorophylls and Carotenoids Content 

Chlorophyll and total carotenoids estimations in plants gave an impression of the physiological condition of these plants. Here, the JN74-OE and control were tested for chlorophyll content and carotenoids under normal conditions in 95% ethanol extraction, according to [34]. Chlorophyll a, chlorophyll b, and carotenoids expressed slight differences in the JN75-OE, but without noticeable significance, as shown in Figure 5c. 

#### 3.4.3. GA_3_ Treatment 

**Figure 6:**
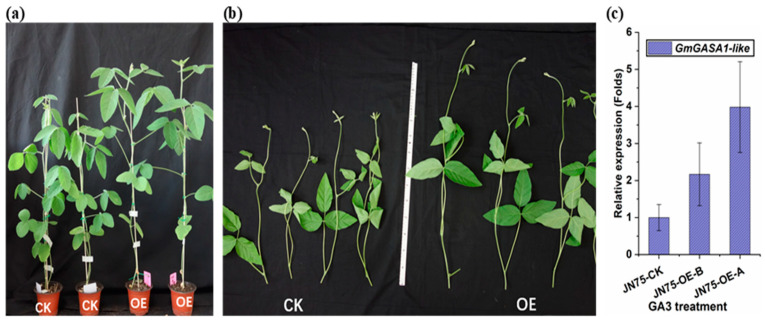
Effect of GA_3_ (0.1 g/L) treatment on T2 generation of JN74-OE vs. CK. (**a**) Plants before treatment at the age of one month. (**b**) Plants 7 days post-treatment. (**c**) The *GmGASA1-like* gene’s relative expression response to GA_3_ treatment after 24 h. Samples for gene relative expression were collected from leaf tissues 24 h after the treatment. Soybean *β-tubulin* (*Glyma20g27280*) gene was used as a reference gene. Error bars represent standard deviation n = 3.

Seven days after the application of GA_3_ solution (0.1 g/L), the JN74-OE and the control plants were monitored. Figure 6b shows shoots that were cut more than 30 cm above the first node in both lines. The plant height of JN74-OE plants significantly increased compared with the control. Also, the internodes of JN74-OE were larger than those of the control when measured longitudinally. Therefore, the higher phenotype of the JN74-OE plants was due to the internode cell enlargement in the stem. The results also showed that *GmGASA1-like* gene expression in JN74-OE was positively regulated by GA_3_ application, increasing from twofold before treatment to fourfold after 24 h compared to the control (Figure 6c).

#### 3.4.4. Cold and Heat Stresses

**Figure 7:**
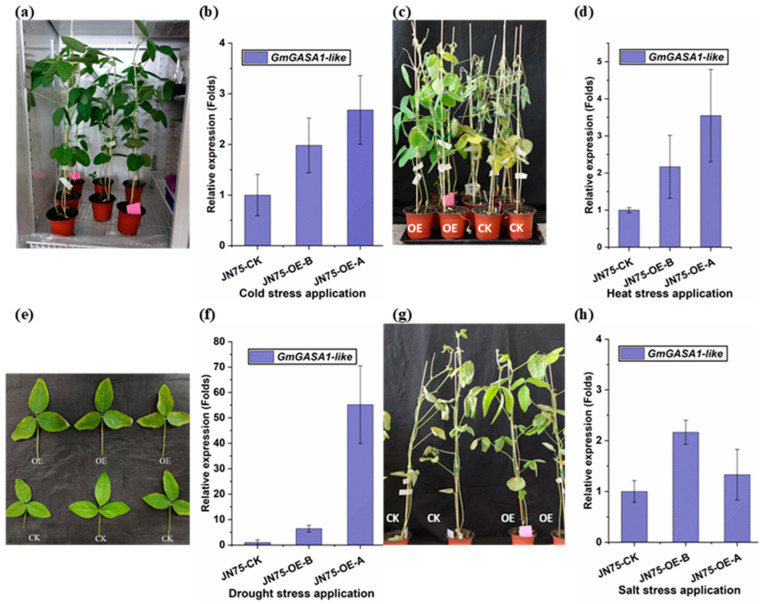
Application of cold, heat, drought, and salt stresses on T2 generation of the JN74-OE vs. CK. (**a**) Plants after 7 days from the cold stress application, at 4 °C. (**b**) Relative gene expression under cold stress. (**c**) Plants after 7 days from the heat stress application, at 42 °C. (**d**) Relative gene expression under heat stress. (**e**) Plants after 7 days from the drought stress application. (**f**) Relative gene expression under drought stress. (**g**) Plants after 7 days from the salt stress application by 250 mM NaCl solution. (**h**) Relative gene expression under salt stress. Samples for gene relative expression were collected from leaf tissues 24 h post-treatment. Soybean *β-tubulin* (*Glyma20g27280*) gene was used as a reference gene. Error bars represent standard deviation; n = 3.

One-month old JN74-OE and the control plants were exposed to 4 °C and 42 °C. After 24 h, leaf tissue samples were collected for the estimation of relative gene expression. Gene expression of the *GmGASA1-like* gene responded positively to cold and heat stresses. In JN74-OE plants, *GmGASA1-like* gene expression increased from twofold to approximately threefold after 24 h of cold stress compared to the control, as shown in Figure 7b. In contrast, gene expression changes before and after 24 h of heat stress ranged from twofold to about fourfold, as shown in Figure 7d. Plants were kept and monitored after 7 days of treatments. No abnormalities were observed in the plants treated at 4 °C (Figure 7a), whereas all plants in heat treatment were dead, except two out of nine pots from the JN74-OE, as shown in Figure 7c. 

#### 3.4.5. Salt and Drought Stresses 

For the drought treatment, JN74-OE plants and control were left without watering until the symptoms of dryness were observed clearly on the leaves’ margins. Then, water was given, and observations were recorded. Leaves in the JN74-OE and control had recovered with little noticeable differences, as shown in Figure 7e. However, JN74-OE exhibited significantly greater levels of gene expression, increasing from twofold to more than fiftyfold compared to the control, both before and after drought stress, just prior to watering for recovery, as shown in Figure 7f. In contrast, exposure to 250 mM NaCl solution resulted in a decrease in gene expression levels in JN74-OE, from 2-fold to about 1.5-fold before and after 24 h of treatment compared to the control, as shown in Figure 7h. Symptoms recorded 7 days after the salt stress treatment showed that all control plants and most of the JN74-OE have died, while the overexpressed lines tolerated the stress longer and died after more days, as shown in Figure 7g. 

#### 3.4.6. Response to *Cercospora sojina* Fungus and the Soybean Mosaic Virus

**Figure 8:**
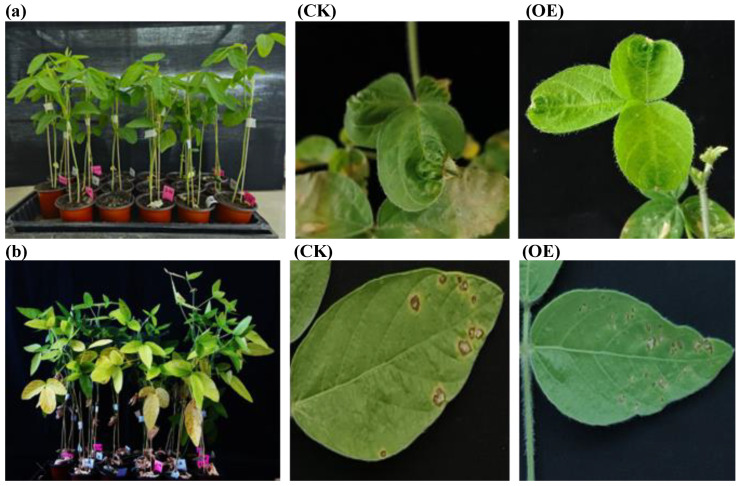
Applied biotic stresses on T2 generation of the JN74-OE vs. CK. (**a**) Symptoms of sensitivity to SMV inoculation are present at the tips of the leaflets in both JN74-OE and CK plants. (**b**) Symptoms of sensitivity to *Cercospora sojina* fungus inoculation were observed in both JN74-OE and CK plants.

Some *GASA* family members have been reported to have a role in plant defence against biotic stresses [44]. Here in this study, two common diseases, the *Cercospora sojina* fungus and the SMV, were subjected to overexpressed and non-transgenic lines to test the resistance probability. After one month of the inoculation treatments, symptoms of infection by the fungus and virus were observed on both lines, the overexpressed and control, as shown in Figure 8. Both JN74-OE and control plants showed susceptibility to the *Cercospora sojina* fungus and the SMV. 

## 4. Discussion

The *GASA* genes generally encode small proteins with three distinct domains: a single peptide domain in the N-terminal sizing between 18–29 residues, a variable region in both length and peptide chain sequences (7–31 polar residues), and the third domain is a segment of approximately 60 amino acids incorporating a conserved domain of 12 cysteine residues named GASA at the C-terminals [16,17]. In soybeans, little information has been released on the *GASA* gene family. In this study, a new member of the soybean *GASA* gene family (*GmGASA1-like* gene) was characterised in silicon and overexpressed in soybean cultivar (JN74) to evaluate the growth, yield, and performance under some main stressors affecting soybeans. 

Promoter analysis of the *GmGASA1-like* gene revealed numerous cis-acting regulatory elements (CAREs). It contained three light-responsive elements: one MRE, one G-box, and two GT1motifs. Additionally, several stress-responsive elements were identified, including an anaerobic-responsive element (ARE), MYB recognition sites for drought and ABA signals, as well as a MYC recognition site for drought, ABA, and cold signals. Hormone-responsive elements, such as the gibberellin-response element (P-box), were also present, suggesting the *GmGASA1-like* gene plays a role in modulating various developmental processes in plants. 

The JN74-OE and control plants were evaluated at the R1-growth stage to observe phenotypic changes across nine growth traits. The JN74-OE plants were significantly taller, had more stem nodes, and exhibited a larger canopy in the field trial compared to the controls. Bioinformatic analysis suggests that this increased height may stem from the interaction of the *GmGASA1-like* gene with gibberellin and other genes involved in cell expansion and division. Similarly, overexpression of the *GmGASA32* gene was found to impact plant height during early growth by interacting with *GmCDC25*, a protein associated with the cell cycle. Activity analysis of the promoter also indicated a response to gibberellin [45]. 

Data collected after harvesting indicated a significant decrease in maximum plant height and the number of nodes per plant in JN74-OE plants compared to controls. However, both traits exhibited an opposite trend during the R1-growth stage. This discrepancy may be attributed to higher yields in overexpressed lines, resulting from an increased number of pods affecting plant height, or to changes in gene behaviour at different growth stages [18]. The seeds’ weight per plant trait showed superiority in JN74-OE plants over the control plants by 5.82 g of seeds as an average for plant yield. The 100 seed weight trait in JN74-OE plants dominated control plants by a 9.8% significant increment. *GASA* family members were reported to influence flowering. Phenotypic analyses indicated that *GASA4* regulates floral meristem identity and positively affects both seed size and total seed yield [46]. In apple trees, *GASA* family members were observed to affect the flowering event through interaction with some main gene family members controlling the flowering process, including *SPL, MADs-box*, and *IDD* gene families [17], which can provide speculation about the reason behind the yield increments. 

In JN74-OE seeds, seed oil content decreased by 3.02%, while seed protein content increased by 2.87%, compared to control plants. These traits are crucial agronomic factors in soybean breeding, often exhibiting a negative correlation [47]. Fatty acid analysis revealed no significant changes in palmitic and linolenic acids, but stearic and oleic acids increased by 9% and 7.39%, respectively, while linoleic acid decreased significantly. Amino acid levels remained largely stable, with notable increases in aspartic acid (0.84%), arginine (1.8%), cysteine (1.85%), glutamic acid (1.14%), and methionine (8.3%). Methionine, a key indicator of soybean protein quality, demonstrated the highest increase.

Data on relative gene expression from soybean germplasms, which exhibit significant differences in plant height, revealed a strong relationship between the plant height trait and the *GmGASA1-like* gene. This correlation is supported by fluctuations in relative expression across phenotypically distinct soybean germplasms, alongside the statistical significance of this relationship. A similar study by Zhang et al. (2012) measured relative expression of the *TaCKX6*-*D1* gene via RT-qPCR in phenotypically diverse rice accessions, finding a significant negative correlation between *TaCKX6-D1* expression levels and the 1000 grain weight phenotype. This suggests that variations in gene expression contribute to observed phenotypic differences [48]. 

Photosynthesis pigments in the JN74-OE and control plants under normal conditions in 95% ethanol extraction were tested according to [34]. Chlorophyll a, chlorophyll b, and total carotenoids were slightly decreased in the overexpressed line without noticeable significance. That slight change may be due to the greater expansion of tissues in the JN74-OE than in the control plants, resulting in lower chlorophyll content in a specific size of leaf in the JN74-OE compared to the control plant. 

GA_3_ application significantly affected the plant height of the JN74-OE plants, more so than that of the control. The increased phenotype was possibly due to the enlargement of internode cells in the stem, in addition to an increase in the number of nodes. The results also showed that *GmGASA1-like* gene expression was positively regulated by GA_3_ application. Factors that affect the biosynthesis of indigenous gibberellin are found to have an impact on *GASA* members, such as the six biosynthetic enzymes that are required for the conversion of geranylgeranyl diphosphate to bioactive GAs or the negative regulators of GA signalling, *DELLAs* [49]. In *Glycine soja*, the overexpression of *GsGASA1* demonstrated inhibition of root elongation, which could result from upregulating some genes (*RGL2* and *RGL3*) among the five *DELLA* genes that regulate the GA signalling pathway [50]. 

In this study, one-month-old JN74-OE and control plants were subjected to cold (4 °C) and heat (42 °C) stresses. After 24 h, *GmGASA1-like* gene expression significantly increased in the JN74-OE plants, rising by more than onefold compared to pre-treatment levels. After 7 days, no abnormalities were observed in the 4 °C-treated plants, while all but two of the heat-treated JN74-OE plants perished. Previous research on *Arabidopsis* indicated that the constitutive expression of *GASA4* enhances heat stress [51] tolerance. Under drought treatment, both plant types showed similar recovery in leaf condition, but JN74-OE plants exhibited gene expression levels exceeding 25-fold just before watering compared to pre-treatment levels. In contrast, exposure to a 250 mM NaCl solution led to reduced gene expression in JN74-OE plants. Seven days post-salt treatment, all control plants perished, while most overexpressed lines survived longer, likely due to interactions with stress-modulating genes. In strawberries, the expression of the *FaGAST2* gene increased under oxidative stress conditions, indicating its role in managing reactive oxygen species [22]. JN74-OE and control plants did not exhibit phenotypic resistance to the following two common diseases: *Cercospora sojina* fungus and SMV. However, some members of the *GASA* family have been reported to play a role in plant defence against biotic stresses, such as the *snakin-2* gene in potatoes [44], *HbGASA7*-1 and *HbGASA15* genes in rubber trees [18], as well *CcGASA4* gene in *Citrus* [52]. 

In summary, the *GmGASA1-like* gene displayed several intriguing aspects related to soybean development, productivity, and environmental adaptability, highlighting its potential role in molecular breeding programmes for this globally important crop. Further research should focus on exploring the relationship between the *GmGASA1-like* gene and other genes involved in flowering, seed pod development, drought resistance, and interactions with GA_3_, where it has shown a notable impact. 

## 5. Conclusions

In this study, we provide new insights into the *GASA* gene family by examining a related member. Our bioinformatic analysis indicates that the *GmGASA1-like* gene is closely linked to the *GA-stimulated AtGASA1* gene from *Arabidopsis*, with its promoter containing several cis-acting regulatory elements (CAREs), including those responsive to hormones. The characteristics of the GmGASA1-like *protein*—its instability, localisation in multiple cellular compartments, abundance of phosphorylation sites, and a single peptide domain—suggest a high potential for activity. The JN74-OE plants exhibited significant changes in plant architecture and positive effects on various seed quality and quantity traits. Their response to key stressors and GA_3_ spraying, along with the expression of the *GmGASA1-like* gene, strongly implicates this gene in soybean adaptability and productivity. While GMOs face restrictions and ongoing controversy in some countries, our research findings contribute valuable knowledge for molecular breeding and soybean research.

## Data Availability

The dataset used in this study is accessible within the article itself and its Supplementary Materials.

## Supplementary Materials

The following supporting information can be downloaded at: https://www.mdpi.com/article/10.3390/life14111436/s1, Figure S1: pMD-18T vector; Figure S2: Positive single colonies and subculture; Figure S3: Comparing sequencing results with *GmGASA1-like* cDNA by DNAman software; Figure S4: pCAMBIAI3301 plant overexpression vector; Figure S5: (a) Double digestion by BstEII and BglII enzymes. (b) Seamless reaction flowchart, https://clonesmart.com/; Table S1: Double digestion of pCAMPIA 3301; Table S2: Seamless assembly reaction; Table S3: Bioinformatic tool used in gene characterisation; Table S4: Used primers for the detection of transgenic plants; Table S5: Used primers for gene relative expression; Table S6: Germplasms of soybean used in testing the relative expression of the GmGASA1-like gene.

## References

[1] Wilson R.F. (2008). Soybean: Market driven research needs. Genetics and Genomics of Soybean.

[2] Ma J., Sun S., Whelan J., Shou H. (2021). CRISPR/Cas9-Mediated Knockout of GmFATB1 Significantly Reduced the Amount of Saturated Fatty Acids in Soybean Seeds. Int. J. Mol. Sci..

[3] Wu N., Lu Q., Wang P., Zhang Q., Zhang J., Qu J., Wang N. (2020). Construction and Analysis of GmFAD2-1A and GmFAD2-2A Soybean Fatty Acid Desaturase Mutants Based on CRISPR/Cas9 Technology. Int. J. Mol. Sci..

[4] Kim D.G., Lyu J.I., Lim Y.J., Kim J.M., Hung N.N., Eom S.H., Kim S.H., Kim J.B., Bae C.H., Kwon S.J. (2021). Differential Gene Expression Associated with Altered Isoflavone and Fatty Acid Contents in Soybean Mutant Diversity Pool. Plants.

[5] Liu K. (2012). Soybeans: Chemistry, Technology, and Utilization.

[6] Liu K., Hettiarachchy N., Kalapathy U. (1997). Soybean protein products. Soybeans: Chemistry, Technology, Utilization.

[7] ASA (2019). A Reference Guide to Soybean Facts and Figures: The American Soybean Association. https://soygrowers.com/wp-content/uploads/2019/10/Soy-Stats-2019_FNL-Web.pdf.

[8] Lu L., Wei W., Tao J.J., Lu X., Bian X.H., Hu Y., Cheng T., Yin C.C., Zhang W.K., Chen S.Y. (2021). Nuclear factor Y subunit GmNFYA competes with GmHDA13 for interaction with GmFVE to positively regulate salt tolerance in soybean. Plant Biotechnol. J..

[9] Schmutz J., Cannon S.B., Schlueter J., Ma J., Mitros T., Nelson W., Hyten D.L., Song Q., Thelen J.J., Cheng J. (2010). Genome sequence of the palaeopolyploid soybean. Nature.

[10] Xu K., Zhang X.-M., Fan C.-M., Chen F.-L., Zhu J.-L., Zhang S.-L., Chen Q.-S., Fu Y.-F. (2017). A callus transformation system for gene functional studies in soybean. J. Integr. Agric..

[11] Hernández S., Franco L., Calvo A., Ferragut G., Hermoso A., Amela I., Gómez A., Querol E., Cedano J. (2015). Bioinformatics and moonlighting proteins. Front. Bioeng. Biotechnol..

[12] Jimenez-Lopez J.C., Gachomo E.W., Sharma S., Kotchoni S.O. (2013). Genome sequencing and next-generation sequence data analysis: A comprehensive compilation of bioinformatics tools and databases. Am. J. Mol. Biol..

[13] Da Sacco L., Baldassarre A., Masotti A. (2011). Bioinformatics tools and novel challenges in long non-coding RNAs (lncRNAs) functional analysis. Int. J. Mol. Sci..

[14] Lei S., Zhao L., Chen Y., Xu G. (2023). Identification and promoter analysis of a GA-stimulated transcript 1 gene from Jatropha curcas. Plant Cell Rep..

[15] Zhang S., Wang X. (2017). One new kind of phytohormonal signaling integrator: Up-and-coming GASA family genes. Plant Signal. Behav..

[16] Aubert D., Chevillard M., Dorne A.-M., Arlaud G., Herzog M. (1998). Expression patterns of GASA genes in Arabidopsis thaliana: The GASA4 gene is up-regulated by gibberellins in meristematic regions. Plant Mol. Biol..

[17] Fan S., Zhang D., Zhang L., Gao C., Xin M., Tahir M.M., Li Y., Ma J., Han M. (2017). Comprehensive analysis of GASA family members in the Malus domestica genome: Identification, characterization, and their expressions in response to apple flower induction. BMC Genom..

[18] An B., Wang Q., Zhang X., Zhang B., Luo H., He C. (2018). Comprehensive transcriptional and functional analyses of HbGASA genes reveal their roles in fungal pathogen resistance in Hevea brasiliensis. Tree Genet. Genomes..

[19] Zhang S., Wang X. (2008). Expression pattern of GASA, downstream genes of DELLA, in Arabidopsis. Chin. Sci. Bull..

[20] Nahirñak V., Rivarola M., Gonzalez de Urreta M., Paniego N., Hopp H.E., Almasia N.I., Vazquez-Rovere C. (2016). Genome-wide analysis of the Snakin/GASA gene family in Solanum Tuberosum cv. Kennebec. Am. J. Potato Res..

[21] Qiao K., Ma C., Lv J., Zhang C., Ma Q., Fan S. (2021). Identification, characterization, and expression profiles of the GASA genes in cotton. J. Cotton Res..

[22] Moyano-Canete E., Bellido M.L., Garcia-Caparros N., Medina-Puche L., Amil-Ruiz F., González-Reyes J.A., Caballero J.L., Munoz-Blanco J., Blanco-Portales R. (2013). FaGAST2, a strawberry ripening-related gene, acts together with FaGAST1 to determine cell size of the fruit receptacle. Plant Cell Physiol..

[23] Achard P., Genschik P. (2009). Releasing the brakes of plant growth: How GAs shutdown DELLA proteins. J. Exp. Bot..

[24] Ahmad M.Z., Sana A., Jamil A., Nasir J.A., Ahmed S., Hameed M.U., Abdullah (2019). A genome-wide approach to the comprehensive analysis of GASA gene family in Glycine max. Plant Mol. Biol..

[25] Tamura K., Stecher G., Kumar S. (2021). MEGA11: Molecular evolutionary genetics analysis version 11. Mol. Biol. Evol..

[26] Li S., Cong Y., Liu Y., Wang T., Shuai Q., Chen N., Gai J., Li Y. (2017). Optimization of Agrobacterium-Mediated Transformation in Soybean. Front. Plant Sci..

[27] Yamada T., Watanabe S., Arai M., Harada K., Kitamura K. (2010). Cotyledonary node pre-wounding with a micro-brush increased frequency of Agrobacterium-mediated transformation in soybean. Plant Biotechnol..

[28] Ma X.J., Fu J.D., Tang Y.M., Yu T.F., Yin Z.G., Chen J., Zhou Y.B., Chen M., Xu Z.S., Ma Y.Z. (2020). GmNFYA13 Improves Salt and Drought Tolerance in Transgenic Soybean Plants. Front. Plant Sci..

[29] Li M., Chen R., Jiang Q., Sun X., Zhang H., Hu Z. (2021). GmNAC06, a NAC domain transcription factor enhances salt stress tolerance in soybean. Plant Mol. Biol..

[30] Shah S.H., Islam S., Mohammad F., Siddiqui M.H. (2023). Gibberellic Acid: A Versatile Regulator of Plant Growth, Development and Stress Responses. Plant Growth Regul..

[31] McDonald S.C., Buck J.W., Li Z. (2023). Pinpointing Rcs3 for frogeye leaf spot resistance and tracing its origin in soybean breeding. Mol. Breed..

[32] Cho E.K., Goodman R.M. (1982). Evaluation of Resistance in Soybeans to Soybean Mosaic Virus Strains1. Crop Sci..

[33] Livak K.J., Schmittgen T.D. (2001). Analysis of relative gene expression data using real-time quantitative PCR and the 2^−ΔΔC(T)^ Method. Methods.

[34] Lichtenthaler H.K., Buschmann C. (2001). Extraction of phtosynthetic tissues: Chlorophylls and carotenoids. Curr. Protoc. Food Anal. Chem..

[35] Pandey V., Krishnan V., Basak N., Marathe A., Thimmegowda V., Dahuja A., Jolly M., Sachdev A. (2018). Molecular modeling and in silico characterization of GmABCC5: A phytate transporter and potential target for low-phytate crops. 3 Biotech..

[36] Manning G., Whyte D.B., Martinez R., Hunter T., Sudarsanam S. (2002). The protein kinase complement of the human genome. Science.

[37] Blom N., Gammeltoft S., Brunak S. (1999). Sequence and structure-based prediction of eukaryotic protein phosphorylation sites. J. Mol. Biol..

[38] Geourjon C., Deleage G. (1995). SOPMA: Significant improvements in protein secondary structure prediction by consensus prediction from multiple alignments. Bioinformatics.

[39] von Heijne G. (1990). The signal peptide. J. Membr. Biol..

[40] Bendtsen J.D., Nielsen H., von Heijne G., Brunak S. (2004). Improved prediction of signal peptides: SignalP 3.0. J. Mol. Biol..

[41] Yang Y., Yu T.F., Ma J., Chen J., Zhou Y.B., Chen M., Ma Y.Z., Wei W.L., Xu Z.S. (2020). The Soybean bZIP Transcription Factor Gene GmbZIP2 Confers Drought and Salt Resistances in Transgenic Plants. Int. J. Mol. Sci..

[42] Lescot M., Déhais P., Thijs G., Marchal K., Moreau Y., Van de Peer Y., Rouzé P., Rombauts S. (2002). PlantCARE, a database of plant cis-acting regulatory elements and a portal to tools for in silico analysis of promoter sequences. Nucleic Acids Res..

[43] Kudełka W., Kowalska M., Popis M. (2021). Quality of soybean products in terms of essential amino acids composition. Molecules.

[44] Balaji V., Smart C.D. (2012). Over-expression of snakin-2 and extensin-like protein genes restricts pathogen invasiveness and enhances tolerance to Clavibacter michiganensis subsp. michiganensis in transgenic tomato (*Solanum lycopersicum*). Transgenic Res..

[45] Chen K., Liu W., Li X., Li H. (2021). Overexpression of GmGASA32 promoted soybean height by interacting with GmCDC25. Plant Signal Behav..

[46] Roxrud I., Lid S.E., Fletcher J.C., Schmidt E.D., Opsahl-Sorteberg H.-G. (2007). GASA4, one of the 14-member Arabidopsis GASA family of small polypeptides, regulates flowering and seed development. Plant Cell Physiol..

[47] Hymowitz T., Collins F., Panczner J., Walker W. (1972). Relationship between the content of oil, protein, and sugar in soybean seed. Agron. J..

[48] Zhang L., Zhao Y.L., Gao L.F., Zhao G.Y., Zhou R.H., Zhang B.S., Jia J.Z. (2012). TaCKX6-D1, the ortholog of rice OsCKX2, is associated with grain weight in hexaploid wheat. New Phytol..

[49] Fukazawa J., Miyamoto C., Ando H., Mori K., Takahashi Y. (2021). DELLA-GAF1 complex is involved in tissue-specific expression and gibberellin feedback regulation of GA20ox1 in Arabidopsis. Plant Mol. Biol..

[50] Li K.-L., Bai X., Li Y., Cai H., Ji W., Tang L.-L., Wen Y.-D., Zhu Y.-M. (2011). GsGASA1 mediated root growth inhibition in response to chronic cold stress is marked by the accumulation of DELLAs. J. Plant Physiol..

[51] Ko C.-B., Woo Y.-M., Lee D.J., Lee M.-C., Kim C.S. (2007). Enhanced tolerance to heat stress in transgenic plants expressing the GASA4 gene. Plant Physiol. Biochem..

[52] Wu T., Cheng C., Zhong Y., Lv Y., Ma Y., Zhong G. (2020). Molecular characterization of the gibberellin-stimulated transcript of GASA4 in Citrus. Plant Growth Regul..

