# Supplementary material for: Functional Characterisation of GmGASA1-like Gene in Glycine max (L.) Merr.: Overexpression Promotes Growth, Development and Stress Responses
Source: Life (Basel). 2024 Nov 6;14(11):1436. doi: 10.3390/life14111436 (PMC11595988; doi:10.3390/life14111436)
Supplement: Supplementary file 1 [file life-14-01436-s001.zip › life-3271356-supplementary.pdf]

Supplementary data for artical

Functional Characterization of *GmGASA1-like* Gene Overexpression in *Glycine max* (L.) Merr.: Implications for Plant Growth, Development, and Stress Responses

Mohamed A. S. Khalifa[1,2], Qi Zhang[1], Yeyao Du[1], Nooral Amin[1], Baozhu Dong[1] and PiwuWang[1,*]

[1] Centre of Biotechnology, Jilin Agricultural University, Changchun 130118, China; mohamed.said@agr.cu.edu.eg (M.A.S.K.); zhangxiaoqi6969@163.com (Q.Z.); du8158152022@163.com (Y.D.); aminagric965@gmail.com (N.A.); 18347513054@163.com (B.D.)

[2] Faculty of Agriculture, Cairo University, Giza 12613, Egypt

* Correspondence: peiwuw@163.com

1. *Cloning of GmGASA1-like (Glyma.06G024500.1) gene*

Gene sequence, transcript, code, and amino acid sequences were obtained from the plant genome database Phytozome 13 (https://phytozome-next.jgi.doe.gov/).

Gene Report for Glyma.06G024500

Genome: Glycine max Wm82.a2.v1 (Phytozome genome ID: 275 • NCBI taxonomy ID: 3847)

Gene Identifier: Glyma.06G024500 (PAC:30550949)

Transcript: **Glyma.06G024500.1**

Location: Chr06:1833924..1835173 forward

```
>G.max Wm82.a2.v1|Glyma.06G024500|Chr06:1833924..1835173 forward (1249bp).
///GCATACTGAAGAGTGAAGAGTTTAAGCAAGCTAACGCTCACTGATTGTTTGTAGTATTATCTTTTGACTATAAATAAAGGCTCCTAG
TTTAACATTCCATGCACTTTTCATTTTCAAGCACAATACCTCTTCCAATCTCTCTTTGCTATGGCTATCTCAAAGCTTCTAGTTGCATCC
CTTCTGGTATCCTTTGTCCTCTTCCATCTCGTGGATGCTGATGACCAATCGGTAATTTAATTAAAGATGCTGCTTCTTCATTTTTATATA
TATTAGTGATTCTTTAATTCTGTATAGTTTAAATAATCCTTGTCACCTTATAATTAAATTACTGTTTTAATATTTAACTAATAACTAATT
TATACATGCTTTTGAGAAACCGCAGGCACACGCACAGACTCAGGGTTCTCTTGTTCAGCATATAGGTATTCCATCAGCATCTGTACGCGC
GATATTTTATTAATTTAAGAACAATTTTTTTTAAAAAAAAACAGTGGGTAATGACAACGAATTACTTGATTTTGAGCATATACTTACTAA
ATTAAGGAAAGGCATATGCTCCCCTGATTTTTTTGGGACAACTCTCTTTAGCTCTGTTCACGTCGTCTAAACCAATTAATATATCACCAA
ATTTTAGAACAGAATTGATGTTAAATGATAAGAAAAGATAATTGTGGCAAGCAAAATGTAATCATCATGTTAGTTTATAATTTTATCAAA
CAGTTTACGCACACCAAAAAATCAATATATTGATTTGATTTAATTGAATTAGATTTTGAACTAATTACTAACTTGAATGATGAATTGATT
TCAGATTGTAATGCTGCATGTGCTGCGAGGTGCCGTTTAGCATCTCGTCAACGCATGTGTCACAGAGCATGTGGAACTTGCTGCAGACGC
TGCAACTGCGTGCCACCGGGAACTTCCGGTAACCAAGAAGTGTGTCCCTGTTATGCCAGTCTCACCACCCACGGTGGCAGACGCAAGTGC
CCTTAATTAGAGTGTGATACCCTTCCAACCCATGCAAGTTATTATATATATGGCCTTGCTTTGCAATCTCGTCATGGTATGTTACGTGCA
ACAAAATGTCAATGTGTTCGAAGACTACCAGCGTCGGTGGAAAATAAACTTGTGTCTTTTCAATGCATGTTGAATCGTTGCAGTGCTATT
ATGCTTTCTCTTTATCAGCTGTTGACTGTTGTATCGATCATCAAGCTTATATAATAATAAAGCTTAATTTGTTGTGTTGCTA///
```

```
>G.max Wm82.a2.v1|Glyma.06G024500.1 (706bp).
///GCATACTGAAGAGTGAAGAGTTTAAGCAAGCTAACGCTCACTGATTGTTTGTAGTATTATCTTTTGACTATAAATAAAGGCTCCTAG
TTTAACATTCCATGCACTTTTCATTTTCAAGCACAATACCTCTTCCAATCTCTCTTTGCTATGGCTATCTCAAAGCTTCTAGTTGCATCC
CTTCTGGTATCCTTTGTCCTCTTCCATCTCGTGGATGCTGATGACCAATCGGCACACGCACAGACTCAGGGTTCTCTTGTTCAGCATATA
GATTGTAATGCTGCATGTGCTGCGAGGTGCCGTTTAGCATCTCGTCAACGCATGTGTCACAGAGCATGTGGAACTTGCTGCAGACGCTGC
AACTGCGTGCCACCGGGAACTTCCGGTAACCAAGAAGTGTGTCCCTGTTATGCCAGTCTCACCACCCACGGTGGCAGACGCAAGTGCCCT
TAATTAGAGTGTGATACCCTTCCAACCCATGCAAGTTATTATATATATGGCCTTGCTTTGCAATCTCGTCATGGTATGTTACGTGCAACA
AAATGTCAATGTGTTCGAAGACTACCAGCGTCGGTGGAAAATAAACTTGTGTCTTTTCAATGCATGTTGAATCGTTGCAGTGCTATTATG
CTTTCTCTTTATCAGCTGTTGACTGTTGTATCGATCATCAAGCTTATATAATAATAAAGCTTAATTTGTTGTGTTGCTA///
```

>G.max Wm82.a2.v1|Glyma.06G024500.1 CDS (303bp).
///ATGGCTATCTCAAAGCTTCTAGTTGCATCCCTTCTGGTATCCTTTGTCCTCTTCCATCTCGTGGATGCTGATGACCAATCGGCACAC
GCACAGACTCAGGGTTCTCTTGTTCAGCATATAGATTGTAATGCTGCATGTGCTGCGAGGTGCCGTTTAGCATCTCGTCAACGCATGTGT
CACAGAGCATGTGGAACTTGCTGCAGACGCTGCAACTGCGTGCCACCGGGAACTTCCGGTAACCAAGAAGTGTGTCCCTGTTATGCCAGT
CTCACCACCCACGGTGGCAGACGCAAGTGCCCTTAA///

>G.max Wm82.a2.v1|Glyma.06G024500.1.p (100 peptides).
///MAISKLLVASLLVSFVLFHLVDADDQSAHAQTQGSLVQHIDCNAACAARCRLASRQRMCHRACGTCCRRCNCVPPGTSGNQEVCPCY
ASLTTHGGRRKCP*///

1.1 Cloning on pMD™18-T vector

Competent cells preparation

1- *E. coli* (5α) had been streaked on LB agar-plate media from a glycerol stock bacterial solution. The next day, a single colony was inoculated into a flask containing 50mL of LB-medium and overnight incubated at 37°C and 170rpm (Starter culture). On the third day, a sub-culture (~500μL) sub-cultured into another incubation flask containing 50mL of LB-medium, incubated at 37°C, and shaken to OD_{600}=0.4 for about (2hr~3hr).
2- Centrifugation was applied at 4000rpm 4°C for 10minutes in sterile falcon (conical) tubes inside the JA-17 rotor; then, the supernatant was disposed of. From this step, everything is kept on ice.
3- Bacterial pellets were resuspended twice in 10-15mL of ice-cold 0.1M$CaCl_2$ for each tube, then centrifugated at 4℃ 4000rpm for 10 minutes.
4- The bacterial suspension was incubated on ice for 10-20 minutes, then centrifuged at 4℃ 4000rpm for 10 minutes. Afterwards, each tube pellet was resuspended in a 4-10mL ice-cold solution prepared from a+b. Where a is (2mL sterile 100% glycerol) and b is (8mL 0.1M $CaCl_2$). Finally, bacterial suspensions were divided into 100μL volumes and stored at -80°C.
5- The transformation efficiency of chemically competent cells was tested by transforming with a plasmid that contains a positive selection marker.

Cloning for the targeted gene was conducted on pMD™18-T, TaKaRa, Japan, ***Figure S1***. According to the company protocol, then single colonies were selected for growing in 5mL LB-medium amended by 5μg ampicillin overnight. PCR validation was conducted by *RV-M13: 5'GAGCGGATAACAATTTCACACAGG3'*and *M13-47:5'GCCAGGGTTTTCCCAGTCACGAC 3'* primers for choosing positive colonies for sequencing, ***Figure S2***. Positive samples were divided into two groups. One copy was sent for sequencing, and another was saved at -80ºC after being mixed with 80% glycerol in a ratio of 7:3 bacterial culture and 80% glycerol for further usage. Sequencing was

done by Comate Bioscience Co., Ltd., China. Results were analysed by DNAman software (https://www.lynnon.com/) by comparing the target gene transcript sequence obtained from the phytozome database (https://phytozome-next.jgi.doe.gov/) with sequencing data, **Figure *S3***. The valid samples were marked for culturing their previous saved sample for further plasmid extraction.

Plasmid Extraction

Cloned *E. coli* by target genes were picked out from -80ºC and grown in LB- media (5µL bacterial solution+ 5µL ampicillin+ 5mL LB-media) overnight at 37ºC and 170rpm. PurePlasmid Miniprep Kit (CW0500S) was used for extracting plasmids and then qualified by Nanodrop1000. High-quality purified plasmids were used for generating the targeted genes with adaptors (S: 5'actcttgaccatggtagatct……… *GmGASA1-like*…….'3 and AS: 5'ggggaaattcgagctggtcacc…… *GmGASA1-like* ………'3) by PCR for further ligations on plant overexpression vector pCAMBIA3301.

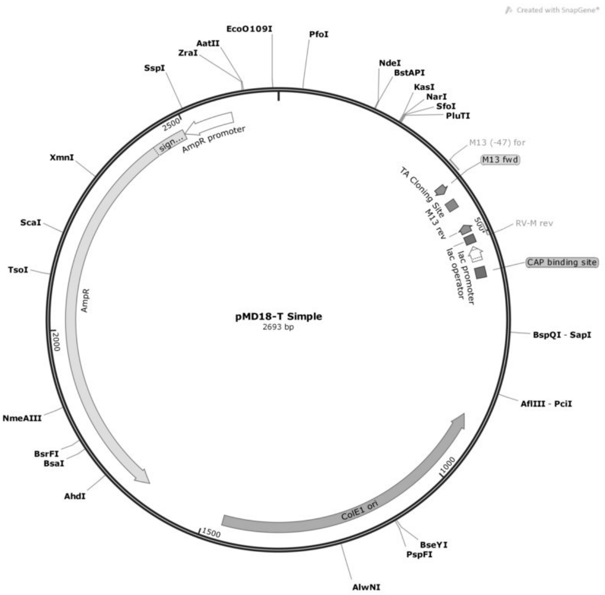

***Figure S1**. pMD-18T vector*

(a) (b)

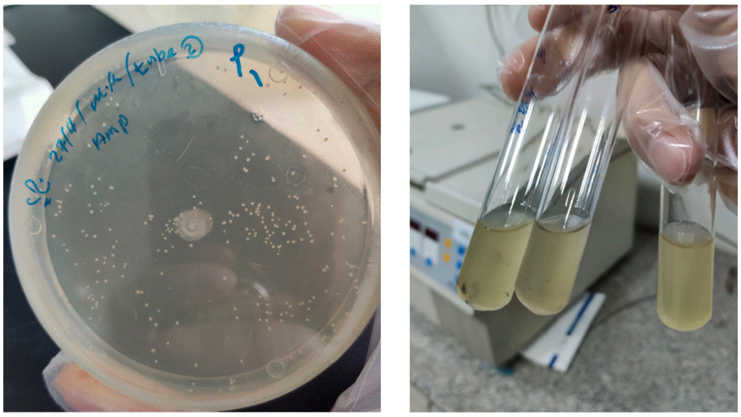

Figure S2. *Positive single colonies and subculture, a and b.*

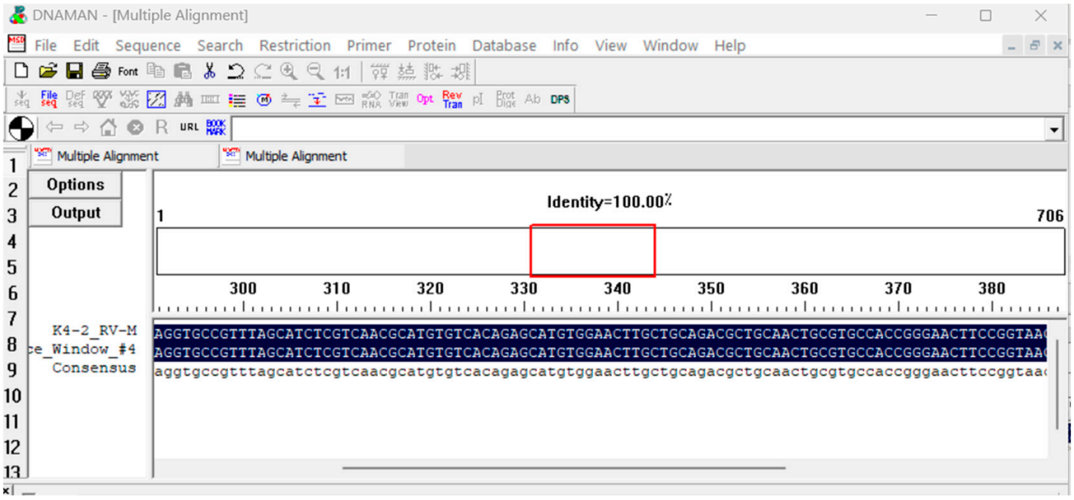

Figure S3. *Comparing sequencing results with GmGASA1-like cDNA by DNAman software*

1.2 Cloning of *GmGASA1-like* on pCAMBIA3301 Vector

pCAMBIA3301 vector was provided from the JLAU biotechnology laboratory in *E. coli* bacterial solution. Bacterial solution was streaked on LB-agar plates with kanamycin antibiotic for single colonies induction, which was cultured later overnight on (5μL bacterial solution+ 5μL kanamycin+ 5mL LB-media) for further plasmid purification. GUS region in the pCAMBIA3301 vector (***Figure S4***) was digested by two enzymes (BglII and BstEII from TaKaRa, Japan) in a PCR reaction, ***Table S1***.

Table S1. *Double digestion of pCAMPIA 3301*

Reaction components	Volume	Digestion temperature
**BstEII**	1μL	37ºC for 2 hour
**BglII**	1μL	60ºC for 1 hour
**10X H Buffer**	2 μL	
**Substrate DNA**	≦ 1 μg	
**Sterile purified water**	up to 20 μL	

Thermal cycles were designed on a PCR machine at 37ºC for 2 hours, 60ºC for 1 hour, and finally, 85ºC for 5 minutes to stop the activity of enzyme residuals so as not to interrupt the next step. The products were electrophoresed over agarose gel for testing quality, ***Figure S5a***.

1.3 Seamless Assembly

After confirming the quality of the digested pCAMBIA3301 vector by BglII and BstEII and preparing the homologous adaptors for the targeted fragments by PCR, the ligation was done by Seamless Assembly Cloning Kit (C5891-25) from Clone Smarter Technologies^{TM}, ***Figure S5b** and **Table 2.***

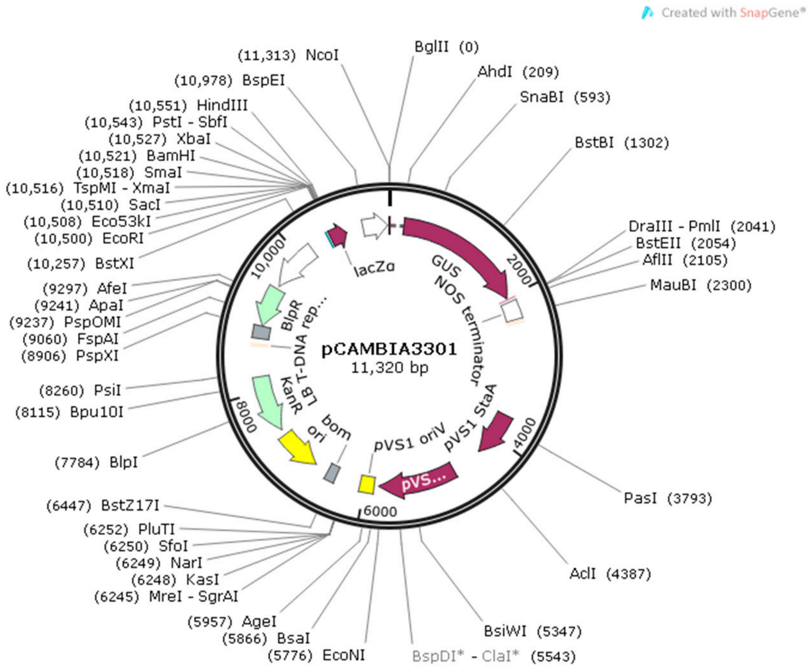

***Figure S4**. pCAMBIAI3301 plant overexpression vector*

***Table S2.** Seamless assembly reaction*

Reaction components	Volume
**DNA fragment (target gene)**	1.5µL
**pCAMBIA3301 backbone**	0.5µL
**Assembly Mix**	5µL
**dd H_2O**	3µL
**Total**	10µL

The percentages between the vector and fragment concentration should be (1:3-10). The following equation was used for that:

$$\frac{vector}{fragment} = \frac{1}{3-10} = \frac{vector(ng) * fragment(bp)}{vector(bp) * fragment(ng)}$$

Where (bp) is the length of DNA and (ng) is the concentration calculated by the nanodrop per 1µL.

The reaction components were incubated at 50ºC for 15 minutes. The cloned vectors were divided into two parts; one part was saved at -20°C, and the other part was used to transform *E. coli*. 5µL of cloned vector plus 100µL competent cells were incubated over ice for 30 minutes afterwards, heat shock at 42°C for 30-60 seconds in a water bath, then quickly over ice for 2 minutes. 1mL of LB-media was add to the mixture in 1.5mL phial and incubated for 40-60 minutes at 37°C. Phials were centrifuged for 5 minutes at 4000rpm (Eppendorf Centrifuge 5810-R). Inside a laminar flow cabinet, 800µL was discarded, and the rest was mixed gently by a 1mL-pipette tip, then spread over LB-kanamycin plates, then incubated at 37°C overnight. Emerged single colonies were grown in (5mL LB-media+5µg kanamycin) overnight incubation at 37°C. The next day, samples were tested by PCR, and the positive samples were confirmed by sequencing. Valid subcultures were used to extract cloned vector (pCAMBIA3301 + *GmGASA1-like*) for transforming *Agrobacterium tumefaciens* strain (AH105), which was provided from JLAU biotechnology centre for *Agrobacterium*-mediated transformation methods using cotyledonary nodes as explants.

(a) (b)

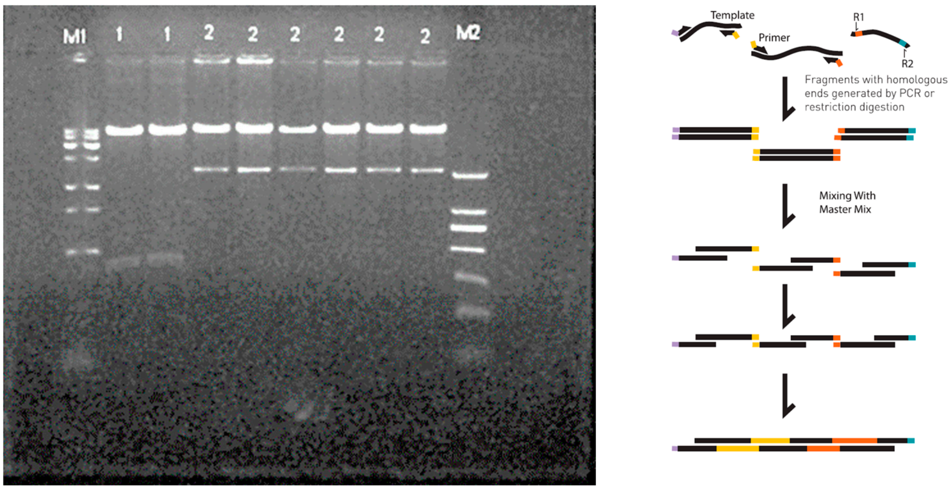

Figure S5. ***(a)***: *double digestion by BstEII and BglII enzymes. M1:12Kpb primer; M2: 2Kpb primer; lanes signed by 1: undigested vector; lanes signed by 2: successful double digested vector.* ***(b)***: *Seamless reaction flowchart,* https://clonesmart.com/

1.4 *Agrobacterium* cloning with target genes

Agrobacterium-competent cells (AH105) were transformed by pCAMBIA3301plasmids harbouring the target gene. Afterwards, PCR reaction with *bar* gene primers (*S: 5'-*

TCAAATCTCGGTGACGGGC -3'; AS:5'- ATGAGCCCAGAACGACGC -3'), and electrophoresis detection were conducted for the single colonies to confirm that *Agrobacterium* harbouring target gene. From the positive cultures some were sent for sequencing and copies were saved at -80°C for further use by adding 300µL (autoclaved 80% glycerol) for 700µL bacterial solution (Hood et al., 1993; Chen et al., 1994; Tiwari et al., 2022). One day before the planned day for infecting explants (co-cultivation step), the saved *Agrobacteria* harbouring the targeted gene were prepared by culturing in 20 mL-test tubes containing 5mL-YEP liquid media amended with 5µg kanamycin antibiotic, overnight incubation at 28°C and 170 rpm. The next day, overnight culture was subcultured in a 10 times volume culture and shaking until the OD reached 0.6-0.8. The OD was determined by using the UVmni-140 spectrophotometer, Shimadzu, Japan. Every culture was transferred to a 50mL falcon tube, sealed with parafilm firmly, and then centrifugated for 5 minutes at 400rpm inside Eppendorf 5810R. Inside the laminar flow, liquid media were discarded, and the bacterial pellets were resuspended in the same size of germination media without agar amended by 1mL/L acetosyringone from stock, which is equal to 200µM. The bacterial suspension was relocated in 200ml autoclaved jars, which were used for shaking the prepared explants for 30 minutes to be infected by *Agrobacterium*. Explants were transferred to filter paper to remove excessive solution and then cultured on co-cultivation media.

2. Characterisation of GmGASA1-like (Glyma.06G024500)

***Table S3**. Bioinformatic tool used in gene characterisation*

Bioinformatic tool	Links
Phytozome database	(https://phytozome-next.jgi.doe.gov/)
PLAZA database,	(https://bioinformatics.psb.ugent.be/plaza/)
andPROTTER	(https://wlab.ethz.ch/protter/start/)
Expasy	(https://web.expasy.org/protparam/)
ProtScale	(https://web.expasy.org/protscale/)
NetPhos-3.1	1 (http://www.cbs.dtu.dk/services/)
SOPMA model	(https://npsa-prabi.ibcp.fr/cgi-bin/secpred_sopma.pl)
AlphaFold	(https://www.alphafold.ebi.ac.uk/)
SignalP 3.0.	(https://services.healthtech.dtu.dk/services/SignalP-3.0/)
TMHMM - 2.0 server	(https://services.healthtech.dtu.dk/service)
ProtComp Version 9.0	(http://www.softberry.com/berry.phtml)
NCBI database	(https://blast.ncbi.nlm.nih.gov/Blast)
MEGA11 software	(https://www.megasoftware.net)
PlantCARE	(https://bioinformatics.psb.ugent.be/webtools/plantcare/html/)
TBtools software	(https://github.com/CJ-Chen/TBtools-II/releases)

3. Primers used for the detection of transgenic plants

Table S4. *Used primers for the detection of transgenic plants*

primer	Sequence	Output band size
35s-F	*5'- TAGAGGACCTAACAGAAC -3'*	500bp
35s-R	*5'- CCGTGTTCTCTCCAAATG -3'*	
NOS-F	*5'- TTTCTTAAGATTGAATCCTGTTGCC -3'*	192bp
NOS-R	*5'- ACCGCGCGCGATAATTT-3'*	
Bar- F	*5'- ATCGTCAACCACTACATCGAGAC -3'*	462bp
Bar-R	*5'- CCAGCTGCCAGAAACCCACGTC -3'*	

4. Primers used for running qPCR experiments

Table S5. *Used primers for gene relative expression*

Primer		Sequence
(*Glyma.06G024500*)	*S*	*5'-GCAAGCTAACGCTCACTGATT -3'*
	AS	*5'-TTGGTCATCAGCATCCACGAG-3'*
Soybean *β-tubulin (Glyma20g27280)*	*S*	*5'- GCTTCGCTGCTCCTGTTACG -3'*
	AS	*5'- GGCTGCTTAACCAATTGCCG -3'*

5. Comparing the gene relative expression in soybean germplasms that are different widely in the plant height trait

Table S6. *Germplasms of soybean used in testing the relative expression of the GmGASA1-like gene*

Short variety	Plant height (cm)	Gene expression in folds	Long variety	Plant height (cm)	Gene expression in folds
**Z083**	48.8	0.32086	**Z232**	114.8	1.686
**Z102**	50.6	0.1428	**Z242**	116	1.369
**Z131**	52.8	0.4328	**Z213**	116.4	0.613
**Z259**	53.5	0.19	**Z224**	117.2	0.626
**Z121**	55	0.2664	**Z190**	118	1.120
**Z118**	56	0.265	**Z198**	120	0.955

Correlation between germplasm height and relative expression of *GmGASA1-like* gene.

Correlations

		GermplasmHeight	GeneRelativeExpression
GermplasmHeight	Pearson Correlation	1	.805**
	Sig. (2-tailed)		.002
	Sum of Squares and Cross-products	12449.663	150.911
	Covariance	1131.788	13.719
	N	12	12
GeneRelativeExpression	Pearson Correlation	.805**	1
	Sig. (2-tailed)	.002	
	Sum of Squares and Cross-products	150.911	2.822
	Covariance	13.719	.257
	N	12	12

**. Correlation is significant at the 0.01 level (2-tailed).

Correlations

		GermplasmHeight	GeneRelative Expression
GermplasmHeight	Pearson Correlation	1	.805**
	Sig. (2-tailed)		.002
	Sum of Squares and Cross-products	12449.663	150.911
	Covariance	1131.788	13.719
	N	12	12
GeneRelativeExpression	Pearson Correlation	.805**	1
	Sig. (2-tailed)	.002	
	Sum of Squares and Cross-products	150.911	2.822
	Covariance	13.719	.257
	N	12	12

**. Correlation is significant at the 0.01 level (2-tailed).
